# Case of Progressive and Morbid Diminution of the Whole Body

**Published:** 1851-10

**Authors:** J. B. Armstrong

**Affiliations:** Cannon Co., Tenn.


*Case of Progressive and Morbid Diminution of the whole Body.* By Dr. J. B. Armstrong, of Cannon Co., Tenn.—June 27th, 1848, I was called to visit Mrs. F., whose case was described by the messenger as being of a consumptive character. When I arrived at her house, I found many of her friends present, anxious to hear the result of my examination. They had been in great doubts whether she was not laboring under consumption. I was informed that she was between the seventh and eighth months of pregnancy. Her friends had been led to fear consumption from the extreme emaciation of her whole system. The general atrophy, or morbid diminution, had gone as far as we frequently find in the last stages of phthisis pulmonalis, and tabes mesenterica. This had occurred in a very gradual manner, from the earliest stage of pregnancy. Her temperament was predominantly nervous. Her tongue was slightly red, and the papillæ somewhat elevated. Pulse 70, and of moderate volume; skin dry and husky; constant thirst for all kinds of drink, and her countenance looked natural and lively. She stated she had not been very sick, and at present was not suffering from any particular pain; but that she had grown so very lean, as to be unable to attend to business of any kind. She was weak when called into exercise, and at the same time it was without any great sense of feebleness.

All the unnatural conditions of the system, as related by her, were dizziness, slight headache, and extreme costiveness, with an ungovernable appetite for all kinds of food, and especially meats. She had indulged her appetite freely, and her digestion appeared good.

I found no unhealthy indications from my examinations over the regions of the thorax and abdomen—except slight tenderness in the epigastric region. Pregnancy rendered my explorations imperfect, as regards the bowels, &c. It was common when in health for her bowels to be evacuated not more than once in seven days, but now it had passed as many as fifteen and eighteen days.

In this case, I found such extreme aberration of the nutritive function, as to excite in my mind the deepest interest. She was a moderate size lady when well, but now would not have weighed more than sixty-five pounds.

The farther my examination was carried, the greater was my astonishment, to find such extreme thinness of the muscular system. I could grasp with perfect freedom many of the organs contained in the abdomen, which were not too near, or behind the womb. Whilst thus seated, and continuing a manual examination, my attention was directed to the womb through the walls of the abdomen. I found it to be equally involved in the emaciation. At this moment the fetus moved, and pressed one foot against the parietes of the uterus, in such a manner as to present to the eye the full shape of the foot through the integuments of the abdomen. Mrs. F. caught hold of its foot, and said in the same way she could often feel of its hand, and distinguish its thumb from its fingers. I did not make the attempt, yet, I think it very probable that I could have carried my examinations so far as to have distinguished the sex of the fetus, and, too, without injury to it, or much pain to the lady.

Probably owing to pregnancy, the abdominal muscles and integuments had not contracted, as we would suppose ordinarily with such general emaciation.

I found so little derangement fixed upon the most important indexes of the system, that it was with great difficulty I could prescribe, with any certainty for her relief. It was already too evident should she continue to go down hill further before the proper period of parturition, she would not be able to bear up under the labor of giving birth to her child.

Consequently, I prescribed for her—1st. Directed a pill of equal parts of colocynth, rhubarb and aloes, purposely to produce more frequent evacuations from the bowels. Next in view, I wished to prescribe a tonic to sustain and prevent further emaciation. In one respect I thought she needed a tonic, and in others it appeared counter-indicated—for this reason, I merely prescribed the compound tincture of gentian, in doses of two fluid drachms three times daily. The cathartic was so regulated as to produce a regular evacuation every forty-eight hours.

On the 30th I visited her again, and found slight improvement, and treatment continued.

On the 10th of July I called in to see her again, and found her, as she supposed, a little stronger. Treatment was continued as before. In this way she continued up to the birth of her child—it occurring in the most natural order. The child supposed to weigh between three and a half and four pounds.

After the first month from the birth of her child, recovered—gaining strength and flesh rapidly, and at the end of four months, she looked to be in common health.

Her husband informs me that she has had but one catamenial discharge since the birth of her child, and he has reason to fear she is now approaching the same form of disease. Should such be the case, what would be the best and most successful mode of treatment?—*Ibid.*

